# Targeted metabolomic profiling of cerebrospinal fluid from patients with progressive multifocal leukoencephalopathy

**DOI:** 10.1371/journal.pone.0242321

**Published:** 2020-11-24

**Authors:** Yi Luo, Nora Möhn, Amani Al-Mekhlafi, Sven Schuchardt, Thomas Skripuletz, Wolfram Sühs, Frank Pessler, Martin Stangel

**Affiliations:** 1 Department of Neurology, Hannover Medical School, Hannover, Germany; 2 Helmholtz Centre for Infection Research, Braunschweig, Germany; 3 Fraunhofer Institute for Toxicology and Experimental Medicine (ITEM), Hannover, Germany; 4 TWINCORE Centre for Experimental and Clinical Infection Research, Hannover, Germany; Heinrich-Heine-Universitat Dusseldorf, GERMANY

## Abstract

Progressive multifocal leukoencephalopathy (PML), caused by JC polyomavirus, is a demyelinating disease of the central nervous system that primarily affects oligodendrocytes. It can cause significant morbidity and mortality. An early diagnosis is of high relevance as timely immune reconstitution is essential. However, diagnosis can be challenging if virus detection via cerebrospinal fluid (CSF) PCR remains negative. Hence, identifying CSF biomarkers for this disease is of crucial importance. We applied a targeted metabolomic screen to CSF from 23 PML patients and eight normal pressure hydrocephalus (NPH) patients as controls. Out of 188 potentially detectable metabolites, 48 (13 amino acids, 4 biogenic amines, 1 acylcarnitine, 21 phosphatidylcholines, 8 sphingolipids, and the sum of hexoses) passed the quality screen and were included in the analyses. Even though there was a tendency towards lower concentrations in PML (mostly of phosphatidylcholines and sphingomyelins), none of the differences between PML and controls in individual metabolite concentrations reached statistical significance (lowest p = 0.104) and there were no potential diagnostic biomarkers (highest area under the ROC curve 0.68). Thus, CSF metabolite changes in PML are likely subtle and possibly larger group sizes and broader metabolite screens are needed to identify potential CSF metabolite biomarkers for PML.

## Introduction

Progressive multifocal leukoencephalopathy (PML) is a rare, opportunistic, demyelinating disease of the central nervous system (CNS) caused by the human JC polyomavirus (HPyV-2). HPyV-2 is a small ubiquitous DNA polyomavirus with a 5.13 Kb circular enclosed double-stranded DNA. Antibodies against this virus are detectable in about half of all people worldwide [1, 2]. In healthy conditions the infection usually does not cause disease. Especially in immune-compromised patients, for example patients suffering from acquired immune deficiency syndrome, or hematologic malignancies, in organ transplant recipients, and patients treated with immunosuppressive drugs the infection with HPyV-2 can cause a progressive demyelination of the CNS. Unfortunately, there is no effective anti-HPYV-2 treatment yet, but immunosuppressants should be discontinued when possible [3–6]. As PML usually occurs as part of the underlying disease, the clinical presentation varies. Besides a clinical neurological examination, brain MRI and cerebrospinal fluid (CSF) analysis are standard procedures in the diagnostic work-up. The detection of HPyV-2 DNA in CSF via quantitative polymerase chain reaction (qPCR) proves the diagnosis of PML. As qPCR results may be negative in early stages of the disease, a negative CSF HPyV-2 PCR does not rule out PML. In cases where ascertaining the diagnosis of PML remains elusive, a brain biopsy with detection of HPyV-2 protein by immunohistochemistry and/or HPyV-2 DNA by in situ hybridization or PCR assay is considered the gold standard for diagnosis and needs to be conducted. However, brain biopsy has several disadvantages such as invasive protocol, risks of complication, and poor patient compliance, which limits its usefulness for dynamic surveillance and follow-up [7–10]. Therefore, novel biomarkers for the diagnosis of PML especially in PCR-negative patients are needed.

As demonstrated in studies on neurodegenerative disorders (for example Alzheimer’s disease or amyotrophic lateral sclerosis) and also in the context of neuroinflammation, metabolomic profiling of CSF has a considerable potential for identifying biomarker candidates and elucidating disease-associated metabolic networks [11–15]. Furthermore, our previous studies have shown that metabolomic analysis of CSF can identify biomarkers that are able to discriminate CNS infections with varicella zoster virus (VZV) infection from non-infectious diseases [16], identify enterovirus infection in patients with normal CSF cell count [17], distinguish between viral and autoimmune neuroinflammation [18], and identify bacterial CNS infections with high sensitivity [19].

In the present study, we applied the AbsoluteIDQ®-p180 kit (Biocrates Life Science AG, Innsbruck, Austria) for CSF analysis as a targeted metabolomics approach based on liquid chromatography tandem mass spectrometry (LC-MS/MS) to investigate differences in CSF metabolic profiles between patients with PML and controls with normal pressure hydrocephalus (NPH). The aim was to identify and validate potential biomarkers for the diagnosis of PML, especially in HPYV-2-PCR negative patients.

## Materials and methods

### Patients and sample preparation

The study subjects comprised 23 patients with PML and 8 patients with normal pressure hydrocephalus (NPH) as controls. All patients were treated at the Department of Neurology at Hannover Medical School during the years 2007 and 2019. CSF samples were obtained via lumbar puncture. The following routine CSF parameters were determined within 2 hours: cell count, lactate concentration, protein concentrations (via Bradford dye-binding assay). HPYV-2 DNA concentrations were measured with a real-time PCR assay. Determination of HPYV-2 DNA concentrations by PCR and HPYV-2 antibody specificity index in CSF were performed as routine tests at the Institute of Virology, Hannover Medical School, Germany, and at the Institute of Virology, University of Düsseldorf, Germany, as described before [20]. qPCR for the detection of HPYV-2-DNA was performed amplifying a 78 bp product in the large T antigen region. For the HPYV-2 antibody specificity index serum and CSF were tested in an enzyme-linked immunosorbent assay using a HPYV-2-VP1 protein fused to GST as antigen, and the HPYV-2 antibody specificity index was calculated. The calculation required the additional determination of albumin and total IgG from serum and CSF. The remaining CSF was immediately centrifuged and the cell-free supernatant frozen in aliquots and stored at −80°C until metabolomic analysis. The PML patients were further divided into two subgroups according to the PCR results: PCR positive PML (PML-P, n = 18) and PCR negative PML (PML-N, n = 5) patients.

The study was approved by the institutional review board of Hannover Medical School (file no. 2413–2014). All patients gave written informed consent to the use of their data.

### Metabolite profiling

#### LC-MS/MS and FIA-MS/MS conditions

All CSF samples were shipped on dry ice and analyzed in duplicate using a targeted metabolomics kit (AbsoluteIDQ®p180 Kit: BIOCRATES Life Sciences AG, Innsbruck, Austria). This approach allows for the simultaneous quantification of 188 metabolites from different compound classes (21 amino acids, 21 biogenic amines, 40 acylcarnitines, 38 acyl/alkyl phosphatidylcholines, 38 acyl/alkyl phosphatidylcholines, 14 lysophosphatidyl-cholines, 15 sphingomyelins, and the sum of hexoses) using a combination of liquid chromatography (Agilent 1290 Infinity II LC, Santa Clara, CA, USA) and mass spectrometry (AB SCIEX 5500 QTrap™ mass spectrometer; AB SCIEX, Darmstadt, Germany). The lipids, acylcarnitines, and the hexoses were determined via flow injection analysis-tandem mass spectrometry (FIA-MS/MS), while the amino acids and biogenic amines were measured by LC-MS/MS. Sample preparation and measurements were performed according to the manufacturers’ instructions. It has to be noted that some of the CSF samples had been stored for more than ten years. Knowledge about possible effects on long-term storage of CSF samples on metabolite profiling is rare. Regarding serum and plasma, metabolomic profiles are affected by repeated freeze/thaw cycles [21], but not by storage time. Additionally, different studies could prove that the levels of CSF biomarkers or the stability of small-molecule metabolites did not significantly change during long-term storage at -80°C [22–24].

#### Data processing

After normalization and pre‐processing of the data, MetIDQ™ software (Biocrates) was used for peak integration and calculation of metabolite concentrations. All analytes that were above the limit of detection (LOD) in ≥50% of patients were selected for further investigation. Values below the LOD were replaced by the reference value provided by the manufacturer (LOD/2), and all values between LOD and LLOQ were replaced by the value LLOQ/2. Nine analytes (citrulline, dopamine, glycine, leucine, methionine, proline, spermidine, t4OH-proline, tryptophan) passed the initial screen based on LOD but were subsequently excluded because most of the values >LOD were <LLOQ. The concentration data were entered into the Metaboanalyst 3.0 software for multivariate analyses, including partial least squares discriminant analysis (PLS-DA), classical univariate receiver operating characteristic (ROC) curve analysis and multivariate ROC curve based exploratory analysis.

### Statistical analysis

The clinical characteristics of all patients and the relative concentrations of the CSF metabolites are presented as mean ± standard deviation (SD). For demographic and clinical characteristics, outcome comparison in PML and control groups was achieved using the independent sample t test for continuous variables and chi-square test for categorical variables. The Wilcoxon Mann-Whitney U test, as an alternative for not normally distributed variables, was applied to determine whether there was a significant difference between the two groups regarding the concentration of each metabolite. P values are 2-tailed and P < 0.05 was considered statistically significant. All analyses were performed using SPSS version 24.0 (IBM, New York, USA).

## Results

### Patients’ characteristics and standard CSF parameters

The demographics and clinical characteristics of the PML subjects are shown in Table 1. Underlying diseases of the PML group included human immunodeficiency virus infection (n = 7), relapsing remitting multiple sclerosis treated with natalizumab (n = 4), lymphoproliferative disorders (n = 6), vasculitis (n = 2), liver transplant (n = 1), kidney transplant (n = 1), bronchial carcinoma (n = 1), and one case with unknown cause of PML (n = 1). There was no significant difference in age or sex between the two groups. Regarding the standard CSF parameters, patients with PML showed a significantly higher level of CSF leukocytes (8 vs 1 per/μL) and CSF protein (624 vs 419 mg/L) compared with NPH patients. This effect was largely due to the HIV patients. Within the PML group, the PCR negative subgroup demonstrated a significantly lower number of CSF leukocytes compared with the PCR positive subgroup (mean 1/μL and 10/μL, respectively). There also was a tendency toward lower CSF lactate (p = 0.239) concentrations in the PCR negative subgroup than the PCR-positive subgroup (Table 2).

**Table 1:** Demographics and clinical characteristics of PML patients.

Patient	Age (years)	Sex (M = male, F = female)	Underlying immune defect	HPYV-2 DNA (copies/mL)
P1	36	M	HIV	Neg
P2	73	F	NHL	Neg
P3	52	M	VA	Neg
P4	72	M	LT	Neg
P5	42	F	RRMS	Neg
P6	73	M	HIV	Pos
P7	62	F	HIV	3000
P8	77	F	B-CLL	250
P9	36	F	RRMS/NTZ	800
P10	32	F	RRMS/NTZ	260
P11	47	M	HIV	400
P12	65	M	T-CLL	203
P13	65	F	KT	500
P14	68	M	Unknow	1000
P15	52	M	VA	30
P16	76	M	NHL	200000
P17	43	M	RRMS/NTZ	500
P18	54	M	MM	179
P19	77	M	BC	100000
P20	55	M	CLL	500
P21	31	M	HIV,HL	1080
P22	54	M	HIV	249
P23	57	M	HIV	800

BC: bronchial carcinoma, B-CLL: B-cell leukaemia, CLL: chronic lymphocytic leukemia, HIV: human immunodeficiency virus, HL: Hodgkin lymphoma, KT: kidney transplant, LT: liver transplant, MM: multiple myeloma, NHL: non-Hodgkin lymphoma, RRMS/NTZ: relapsing remitting multiple sclerosis treated with natalizumab, T-CLL: T-cell leukaemia, VA: vasculitis. P1 and P4: PML verified by brain biopsy; P2: first HPYV-2 PCR positive, follow up CSF HPYV-2 DNA negative; P3: HPYV-2 PCR positive in follow-up CSF analysis three weeks later; P5: elevated HPYV-2 antibody specificity index. Sample P15 was thawed and re-frozen twice.

**Table 2:** Demographic and CSF characteristics of PML patients compared with NPH patients.

	Control		PML	
		ALL	PCR-positive	PCR-negative
	(NPH = 8)	(n = 23)	(n = 18)	(n = 5)
Age (years)	62.8±15.9	56.5±15.0	56.9±14.9	55.0±17.0
Sex (M/F)	4/4	16/7	13/5	3/2
CSF_leukocytes (per/μL)	1±1	8±13^a^*	10±14^b^*	1±1
CSF_protein (mg/L)	419±141	624±271^a^*	638±281	573±252
CSF_lactate (mmol/L)	1.6±0.2	1.8±0.3	1.8±0.3	1.6±0.2

Data presented as means ± SD, M: male, F: female, CSF: cerebrospinal fluid, NPH: normal pressure hydrocephalus, PML: Progressive multifocal leukoencephalopathy,

^a^: PML vs Control;

^b^: PCR-positive vs PCR-negative,

* p < 0.05.

### Efficiency of CSF metabolite detection via mass spectrometry

Of the 188 potentially detectable metabolites, 48 passed the 2-step quality assessment mentioned above. These metabolites comprised 13 amino acids, 4 biogenic amines, 1 acylcarnitine, 21 phosphatidylcholines, 8 sphingolipids, and the sum of hexoses.

### Metabolomic difference between PML and NPH patients

**Fig 1:**
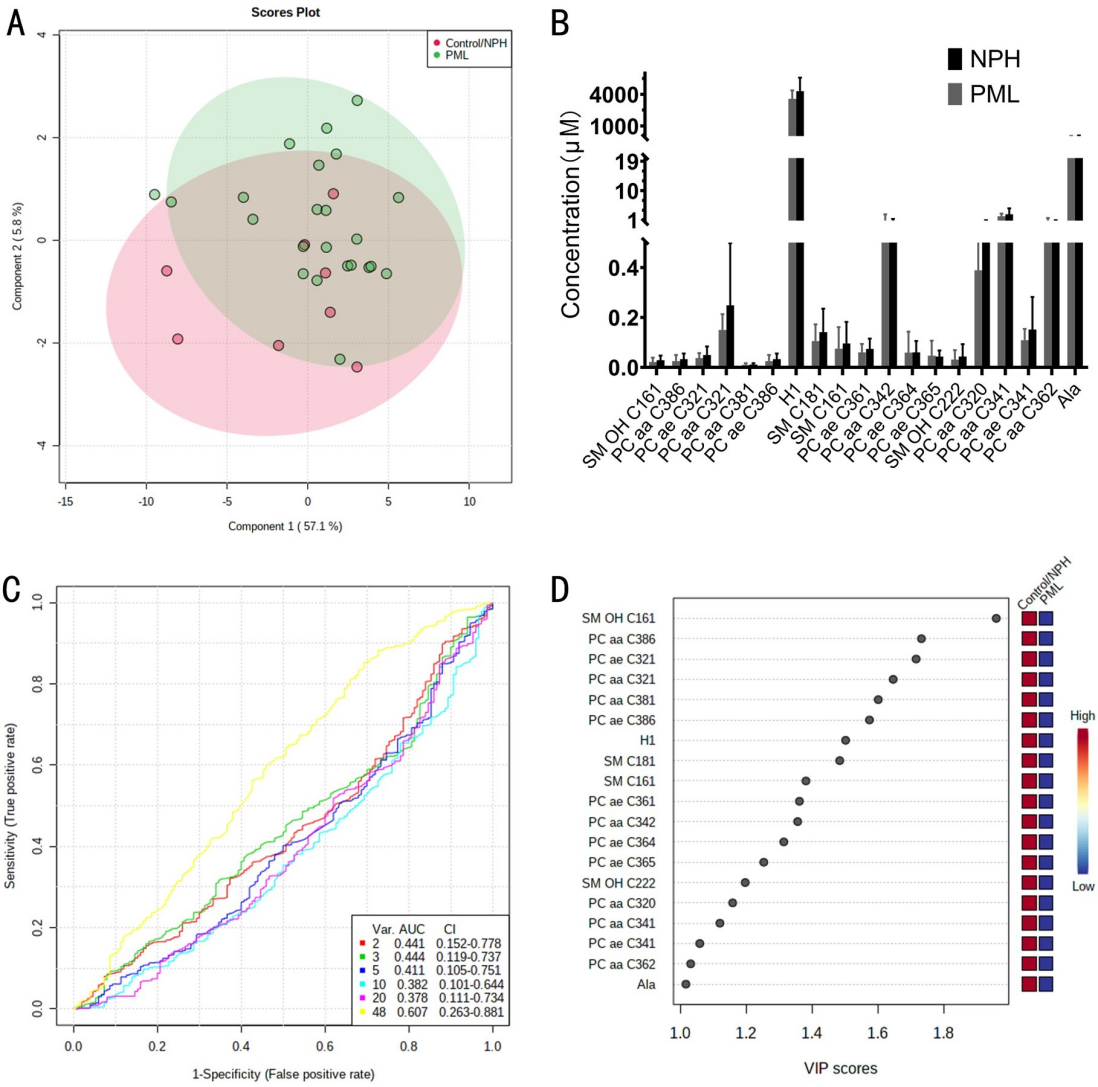
Analysis of metabolomic alterations of the PML and control samples. (A) Partial least squares discriminant analysis (PLS-DA) scores plot showing some separation between the PML (n = 23) and NPH (n = 8) subjects based on their metabolic profile of selected metabolites. (B) Concentrations of the 19 most promising metabolites (VIP>1). (C) Areas under the receiver-operating characteristic curves (AUROCs) of combinations of metabolites. (D) Variable influence on projection (VIP) plot showing the metabolites that are most important in driving the separation of the two groups.

All 48 detected metabolites were analyzed by multivariate statistical analysis, using PLS-DA to identify differentially abundant metabolites. The PLS-DA score plot suggested that there were some differences between PML and NPH metabolite abundance patterns (Fig 1A). Metabolites that could potentially contribute to the separation between PML and NPH patients were, therefore, identified based on a threshold of variable importance in projection (VIP) values (VIP>1.0) taken from the PLS-DA model. Firstly, the 19 metabolites with VIP>1.0 were selected as they seem to be the most influential variables that could separate the two groups (Fig 1C and 1D). These 19 metabolites comprised 13 phosphatidylcholines (PCaaC386, PCaeC321, PCaaC321, PCaaC381, PCaeC386, PCaeC361, PCaaC342, PCaeC364, PCaeC365, PCaaC320, PCaaC341, PCaeC341, and PCaaC362), 4 sphingolipids (SMOHC161, SMOHC222, SMC181, and SMC161), 1 amino acid (alanine), and the sum of hexoses (H1). In the PML group, concentrations of 10/13 phosphatidylcholines, all 4 sphingolipids, alanine, and the generic hexose appeared to be lower than in the control group (Fig 1C). However, subsequent Wilcoxon Mann-Whitney U testing did not reveal statistical significance in any of these metabolites (range of p values: 0.104–0.964). ROC analysis was used to evaluate their discriminatory potential in the binary comparison. As shown in Fig 1B, these metabolites did not exhibit biomarker potential, as AUCs of individual metabolites ranged between 0.5 and 0.679, AUCs of various combined classifiers ranged between 0.411 and 0.607, and all CI crossed the chance line of 0.5.

**Fig 2:**
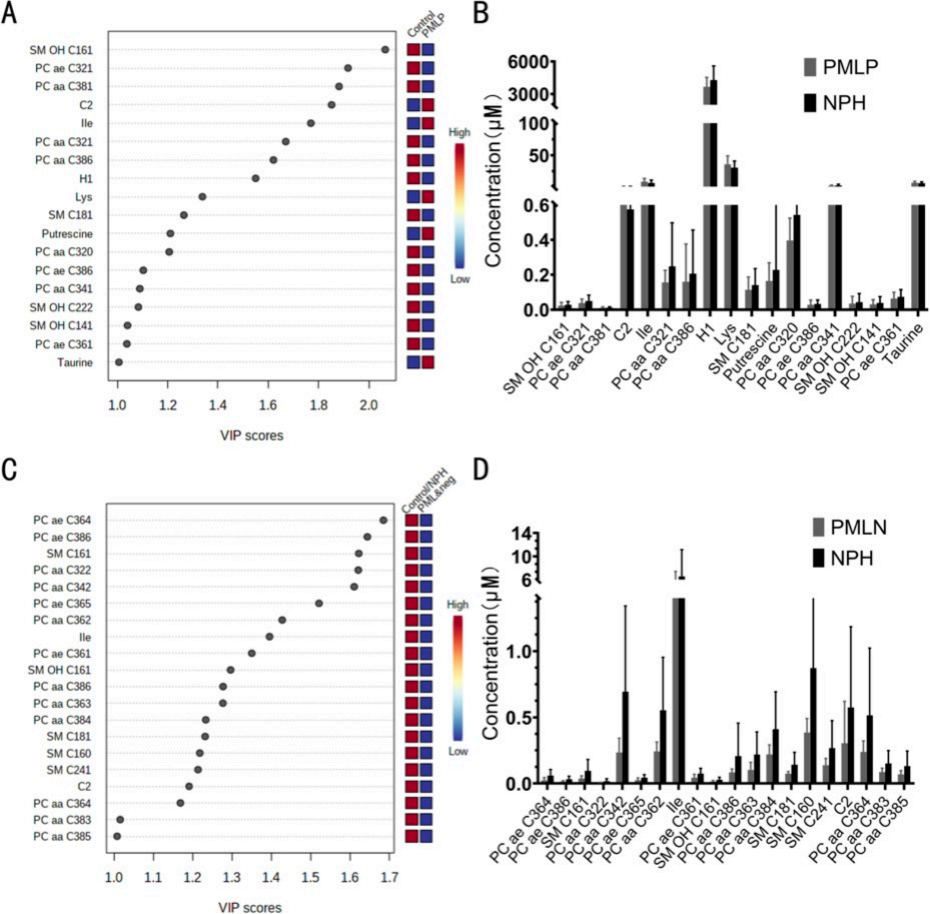
Analysis of metabolomic alterations of the PMLP or PMLN vs. control samples. (A) Variable influence on projection (VIP) plot showing the metabolites that are most important in driving the separation of the PML PCR-positive (PMLP) vs. the NPH group. (B) Concentrations of the 18 most promising metabolites (VIP>1 for PMLP vs. NPH)(C) Variable influence on projection (VIP) plot showing the metabolites that are most important in driving the separation of the PML PCR-negative (PMLN) and the NPH group. (D) Concentrations of the 20 most promising metabolites (VIP>1 for PMLN vs. NPH).

A similar analysis was performed with the PML group being divided into HPYV-2 PCR positive (PML-P) and HPYV-2 PCR negative (PML-N) patients. Using PLS-DA, compared to the control group, we found 18 metabolites with VIP scores ≥1.0 in PML-P patients and 20 in PML-N patients. In the subsequent non-parametric test and ROC curve analysis all of them failed to meet the criteria for significance or biomarker potential (Fig 2).

## Discussion

In this study, we employed a targeted LC-MS/MS approach to investigate the metabolic profile of CSF samples from PML patients in comparison to a control group. CSF is an important source of biomarkers for neurodegenerative and neuroinflammatory diseases because it has close contact to the damaged tissue. Therefore, metabolic changes within the brain are likely to be reflected in the CSF composition [25, 26].

Although the CSF routine examination revealed that the CSF cell count and the CSF protein concentrations in PML patients were significantly higher than those in control patients, this was mostly driven by patients with HIV as the underlying cause for immunodeficiency. This only reflects that an elevated cell count or protein concentration in the CSF does not exclude PML and that the routine CSF parameters are normal in the majority of PML cases. Thus, there is a need for new biomarker for PML, in particular in patients with negative HPYV-2 PCR.

As the CNS contains a high concentration of lipids, it is assumed that metabolites belonging to lipid/fatty acids, glutathione, and energy metabolism have a strong association with autoimmune or degenerative neurological diseases, such as multiple sclerosis or Parkinson’s disease [27–29]. To our knowledge, no metabolomics study in PML using LC-MS/MS has been reported as of yet. Using the PLS-DA analysis (VIP>1.0), we found 19 metabolites that contributed to differences between PML and NPH, which was used as a non-inflammatory/non-infected control group. Even though we did not identify any single significantly differentially abundant metabolite, we did observe a tendency toward lower concentrations (mostly in phosphatidylcholines and sphingomyelins) in the PML group which was accompanied by a tendency toward higher lactate levels. The tendency towards downregulation may be due to a loss of metabolites caused by dysfunction of neurons and/or oligodendrocytes. It has been previously reported that phosphatidylcholine constitutes the backbone of the neural membrane. Sphingolipids are cell membrane-derived lipids which act as signaling molecules and play a critical role in cell death and survival, proliferation, recognition, and migration. The decrease of them is closely related to neuronal pathways involved in neurodegenerative diseases [30, 31]. Those findings are consistent with our current results. However, the metabolites were not statistically significant in further non-parametric and ROC curve analysis and consequently could not serve as diagnostic biomarkers. Also, by combining several metabolites no reliable set of biomarkers could be found to differentiate between PML and controls (see public repository). Taken together, these results suggest that metabolic changes in the CNS of PML patients are relatively subtle. They may be driven by an attenuation of neuronal function due to loss of oligodendrocyte function that is typical of PML [32, 33], which would be consistent with the insidious onset and slow progression of this disease and the lack of overt tissue destruction. In contrast, we have observed pronounced increases in membrane phospholipids in CSF from patients with both viral and bacterial CNS infections that feature an acute clinical course and pronounced neuroinflammation [16–19].

This study has some limitations. Firstly, it captured only a fraction of the more than 430 metabolites that have been reported for CSF [34], and we have no explanation why we detected substantially fewer metabolites >LOD than in our previous studies [16, 17]. It has not yet been shown that long-term storage has a negative influence on the stability of small-molecule metabolites. Secondly, the sample size was relatively small; due to the low degree (or absence) of neuroinflammation a higher sample size may be needed to reach statistical significance than in our previous studies of acute viral CNS infections, where we identified highly accurate biomarkers with even smaller sample sizes [16, 17]. We performed a power calculation to estimate the size of a future cohort study that would be required to expect significance of the two metabolites with the highest AUCs. Assuming equal prevalence of cases (PML) and controls, and correcting for multiple testing of 48 hypotheses, the number of participants (samples) in each group would be 44 to 45 to validate H1 (the sum of hexoses, which is mostly glucose in CSF, AUC 0.70) and 49 to validate PC.aa.C32.2 (AUC 0.69). However, metabolites with AUCs in this range will unlikely prove to be clinically useful biomarkers, and recruitment of larger cohorts is difficult in a rare disease like PML. This analysis therefore suggests that a more promising strategy would be to perform new screens featuring additional classes of molecules in order to obtain more accurate biomarker candidates. Thirdly, it is possible that (even though none of the samples featured pleocytosis) the control samples from NPH patients do not entirely reflect findings in CSF from healthy individuals.

## Conclusion

Mass spectrometry is a key technique in CSF biomarker research, involved in discovery, verification, validation and the establishment of reference methods, and it should not be abandoned as a tool for biomarker discovery for PML. Our results suggest that any changes in CSF metabolites in PML may be subtle and that larger sample sizes and broader metabolite screens are needed to identify clinically useful CSF metabolite biomarkers for this rare and insidious disorder.
